# Siglec15/TGF‐β bispecific antibody mediates synergistic anti‐tumor response against 4T1 triple negative breast cancer in mice

**DOI:** 10.1002/btm2.10651

**Published:** 2024-03-11

**Authors:** Limei Shen, Alison M. Schaefer, Karthik Tiruthani, Whitney Wolf, Samuel K. Lai

**Affiliations:** ^1^ Division of Pharmacoengineering and Molecular Pharmaceutics Eshelman School of Pharmacy, University of North Carolina Chapel Hill North Carolina USA; ^2^ Department of Biomedical Engineering University of North Carolina Chapel Hill North Carolina USA; ^3^ Department of Immunology and Microbiology University of North Carolina Chapel Hill North Carolina USA

**Keywords:** immunotherapy, tumor microenvironment

## Abstract

An ideal tumor‐specific immunomodulatory therapy should both preferentially target the tumor, while simultaneously reduce the immunosuppressive environment within the tumor. This guiding principle led us to explore engineering Siglec‐15 (S15) targeted bispecific antibody (bsAb) to enhance therapy against triple negative breast cancer (TNBC). S15 appears to be exclusively expressed on macrophages and diverse tumor cells, including human and mouse 4T1 TNBC. TGF‐β is a growth hormone frequently associated with increased tumor invasiveness, including in TNBC. Here, to overcome the immune‐suppressive environment within TNBC tumors to enable more effective cancer therapy, we engineered a bispecific antibody (bsAb) targeting both Siglec15 and TGF‐β. In mice engrafted with orthotopic 4T1 tumors, S15/TGF‐β bsAb treatment was highly effective in suppressing tumor growth, not only compared to control monoclonal antibody (mAb) but also markedly more effective than mAbs against S15 alone, against TGF‐β alone, as well as a cocktail of both anti‐S15 and anti‐TGF‐β mAbs. We did not detect liver and lung metastasis in mice treated with S15/TGF‐β bsAb, unlike all other treatment groups at the end of the study. The enhanced anti‐tumor response observed with S15/TGF‐β bsAb correlated with a less immunosuppressive environment in the tumor. These results underscore S15‐targeted bsAb as a promising therapeutic strategy for TNBC, and possibly other S15 positive solid tumors.

**Translational Impact Statement**

This study investigates a bispecific antibody targeting both Siglec‐15 and TGF‐β, demonstrating the benefit of simultaneous targeting of the tumor and immunosuppressive factors within the tumor microenvironment. These results indicate this bispecific antibody as a promising strategy for enhancing therapy against triple negative breast cancer.

## 1 INTRODUCTION

The tumor microenvironment (TME) is comprised of tumor cells and various tumor‐associated cell types, all of which are capable of secreting or expressing various signaling molecules. This includes those targeting immune checkpoints that suppress immune responses within the TME, thereby allowing tumors to evade immune response.^1^ For instance, the overexpression of PD‐L1 and CTLA4 in select tumors can lead to dominant immunosuppressive phenotype in the TME; thus, blocking these pathways is thought to make the tumors more permissible to T‐cells mediated killing.^2^, ^3^, ^4^ Not surprisingly, immunomodulatory agents, such as those that block either the PD‐1/PD‐L1 or CTLA4 pathways, have been extensively investigated as cancer therapies,^2^, ^4^, ^5^ including ipilimumab for melanoma to supplement surgery, radiotherapy, and chemotherapy.^6^ Nevertheless, many patients failed to respond to PD‐1/PD‐L1 or CTLA4 targeted immunomodulatory therapies in clinical trials.^7^, ^8^ This is likely reflective of the inherent genomic instability of cancer cells that create diverse mechanisms to evade immune response or immunotherapy, and underscores the challenge in achieving effective immunomodulation in the TME.

In searching for other immunosuppressive pathways that may be active in PD‐1/PD‐L1 resistant patients, we were drawn to a landmark study by Lieping Cheng's group that showed, using Siglec‐15 (S15) deficient mice, that S15 can suppress T‐cell proliferation and activation in vitro and in vivo.^9^ S15, is a member of sialic acid‐binding immunoglobulin‐like lectins family; while responsible for osteoclast differentiation and bone remodeling,^10^, ^11^ its precise immunological function remains largely unknown. S15 is selectively expressed on a subset of myeloid lineage cells, on tumor cells, and/or on tumor‐associated stromal cells like tumor‐associated macrophages from non‐small cell lung carcinoma clinical samples. S15 appears to be otherwise generally absent in other immune cells as well as healthy tissue across the body.^9^ Interestingly, the immunosuppressive mechanism of S15 appears to be independent of PD‐L1,^9^, ^12^ implicating S15 as a potential immune evasion mechanism in the TME of PD‐L1 negative patients.

In addition to the aforementioned PD‐1/PD‐L1 axis and the CTLA‐4 ligand interactions, there are numerous other immunosuppressive entities in the TME, including regulatory T cells (Tregs), myeloid derived suppressor cells (MDSCs), as well as cytokines such as interleukin‐10 (IL‐10) and transforming growth factor‐β (TGF‐β), all of which could lead to suboptimal anti‐tumor responses.^13^, ^14^, ^15^, ^16^ TGF‐β is a well‐studied pleiotropic cytokine that has a dual role in cancer progression. While it has multiple normal cellular functions, including effects on the cell cycle, motility, angiogenesis, and suppression of the initiation of some tumor types,^17^ TGF‐β can also serve as a driver of tumor progression by suppressing the host antitumor immune response, and by inducing tumor cell plasticity, a process rendering epithelial tumors more mesenchymal, stem‐like and resistant to immune and other therapies.^17^, ^18^ Unfortunately, prior and ongoing clinical trials that sought to inhibit TGF‐β, using either small molecule inhibitors of the serine/threonine kinase domain of the TGF‐β receptor I (TGF‐βRI)^19^, ^20^, ^21^ or anti‐TGF‐β neutralizing mAbs, have enjoyed limited success, leading to doubts about whether TGF‐β is a useful target for cancer therapies.

Guided by these insights, we hypothesized that we could enhance the delivery of anti‐TGF‐β moieties into the TME by coupling it with anti‐S15 moieties that could more specifically bind and accumulate in S15‐positive tumors. Furthermore, we hypothesized that simultaneously targeting both S15 and TGF‐β would more effectively overcome the innate immune‐suppressive environments of the tumors, leading to more effective cancer therapy. These two hypotheses motivated us to engineer a bispecific antibody (bsAb) that can target both Siglec15 and TGF‐β, and evaluate whether co‐inhibition of S15 and TGF‐β could synergistically enhance immunotherapy against triple‐negative breast cancer (TNBC).

## 2 MATERIALS AND METHODS

### 2.1 Bispecific antibody design and production

We engineered various mAbs, including the bsAb that binds both Siglec15 and TGF‐β (abbreviated ST), an IgG mAb that binds S15 only (abbreviated S), and an IgG mAb that binds TGF‐β only (abbreviated T). The mAbs utilized variable region sequences from antibody clones 1H3 (WO2018057735A1) and 2G7,^22^ and contained OrthoMab point mutations introduced to the heavy and light chain interface that enable >90% correct pairing of the two Fabs.^23^ The amino acid sequences were converted to human codon optimized DNA sequence; the Fab domain in the bsAb was linked by a flexible (GGGGS)6 linker in the IgG‐Fab format. The DNA was synthesized through Twist Biosciences, and cloned into mammalian expression vector pαH for secretion in Expi293 cells. The bsAb heavy and light chain DNA was transfected in a 1:2 ratio using manufacturer protocols using expifectamine (Thermofisher) and the supernatant was collected after the cell viability dropped below 60% and purified using Protein‐A/G affinity chromatography using FPLC and purity was verified by SDS–PAGE.

### 2.2 Binding affinity measurement

The binding affinity of the ST bsAb to TGFβ (Acrobiosystems) and Siglec15 (Acrobiosystems) were measured by ELISA. Briefly, half‐area polystyrene plates (CLS3690, Corning) were coated with 50 μL of 2 μg/mL of either TGFβ or S15 in NaHCO_3_ buffer (pH 9.6) and incubated overnight at 4°C. The plate was washed 3× with PBS, followed by incubation with 150 μL of 5% milk for 1 h at room temperature. Following another 3× wash with PBS, serial dilutions of ST IF in 1% milk were added in triplicates to the microwells and allowed to incubate for 1 h at room temperature. The wash procedure was then repeated, followed by addition of 50 μL secondary antibody at 1:10,000 dilution in 1% milk (Goat F(ab′)2 Anti‐Human IgG Fc (HRP), ab98530, Abcam) for 1 h at room temperature. The washing procedure was repeated and 50 μL of the buffer containing substrate (1‐Step Ultra TMB ELISA Substrate, Thermo Scientific) was added to develop the colorimetric reaction for 15 min. The reaction was quenched using 50 μL of 2N H_2_SO_4_, and the absorbance at 450 nm (signal) and 570 nm (background) was measured using SpectraMax M2 Microplate Reader (Molecular Devices).

To measure simultaneous binding, a sandwich ELISA with biotin‐streptavidin detection was used. Plates were coated with 50 μL of 2 μg/mL of TGFβ in NaHCO_3_ buffer and incubated overnight at 4°C. Washes were performed 3× after each incubation step using PBS. The plate was blocked with 150 μL of 5% milk for 1 h at room temperature. Serial dilutions of ST IF in 1% milk were added in triplicates to the microwells and allowed to incubate for 2 h at room temperature. S15‐biotin (Biotinylated Human Siglec‐15, Avitag, SG5‐H82E9, Acrobiosystems) at a 1:5000 dilution in 1% milk was added and incubated for 1 h at room temperature. Finally, streptavidin‐HRP (STN‐NH913, Acrobiosystems) was added to each well at a 1:10,000 dilution in 1% milk. After a final round of washing, 50 μL of the buffer containing substrate (1‐Step Ultra TMB ELISA Substrate, Thermo Scientific) was added to develop the colorimetric reaction for 15 min. The reaction was quenched using 50 μL of 2N H_2_SO_4_, and the absorbance at 450 nm (signal) and 570 nm (background) was measured using SpectraMax M2 Microplate Reader (Molecular Devices).

### 2.3 Cell line and cell culture

Mouse 4T1 TNBC was obtained from tissue culture facility in UNC. The 4T1 cells were maintained in Dulbecco's Modified Eagle Medium: Nutrient Mixture F‐12 (DMEM/F12) supplemented with 10% fetal bovine serum (FBS) (Gibco) and 1% penicillin/streptomycin at 37°C and 5% CO_2_ in a humidified atmosphere.

### 2.4 4T1 TNBC orthotopic model

Six‐week‐old female BALB/c mice were purchased from Charles River Laboratories and maintained under pathogen‐free conditions. All animal‐handling procedures were approved by the Institutional Animal Care and Use Committee at University of North Carolina at Chapel Hill. To establish the orthotopic tumor model, 10^6^ 4T1 TNBC cells suspended in PBS were injected at the fourth nipple of each mouse, and tumor growth was monitored every 2 days by caliper and calculated as (*L* × *W*2)/2, where “*L*” represents the longer dimension and “*W*” represents the shorter one. Treatments were initiated when the tumors reached 100–150 mm^3^ (typically between days 10 and 14 post inoculation), and administered a total of three times given every other day. Before the first treatment, mice were randomized into different groups (*n* = 8–10 per group) receiving the following interventions: control IgG, ST bsAb, S + T mAb, S mAb only, and T mAb only. The treatment doses and volumes are as follows: ST bsAb (200 μg of bsAb in 100 μL), cocktail of anti‐S15 and anti‐TGFβ mAbs (100 μg of anti‐S15 mAb and 100 μg of anti‐TGFβ mAb in 100 μL), and anti‐S15 or anti‐TGFβ mAb (100 μg of mAb in 100 μL).

### 2.5 Flow cytometry assay

Tumor‐infiltrating immune cells were analyzed by flow cytometry. In brief, tumor tissues were harvested and digested with collagenase A and DNase at 37°C for 60 min. After lysis of red blood cells, cells were resuspended in 5 mL of PBS. 2 × 10^6^ cells were stained with various fluorescently labeled antibodies (Table 1) for surface marker expression analysis. After staining, cells were fixed with 300 μL 4% PFA, and analyzed via FACS (BD LSR II).

**TABLE 1:** Fluorescently labeled antibodies used in this study.

Antibody	Clone	Fluorochrome	Catalog number	Company
CD3 (anti‐mouse)	145‐2 C11	PE	553063	BD Biosciences
CD4 (anti‐mouse)	RM4‐5	APC	170042‐82	Invitrogen
CD8a (anti‐mouse)	53‐6.7	Fitc	553030	BD Biosciences
CD11c (anti‐mouse)	N418	Alexa Fluor 594	117346	Biolegend
CD19 (anti‐mouse)	6D5	APC	115512	Biolegend
CD45 (anti‐mouse)	30‐F11	Alexa Fluor 594	103144	Biolegend
B220 (anti‐mouse)	RA3‐6B2	Pacific Blue	558108	BD Pharmnigen
CD69 (anti‐mouse)	H1.2F3	eFluro 450	48‐0691‐82	Invitrogen
CD19 (anti‐human)	HIB19	PE	12‐0199‐42	eBioscience™
CD14 (anti‐human)	61D3	APC‐Cy7	A15453	BD
CD3 (anti‐human)	OKT3	Per‐CP	45‐0037‐42	Invitrogen
Anti‐Siglec 15		Unconjugated	Ab198684	Abcam
Anti‐mouse/human S15 antibody	MK4.5	Purified	164602	Biolegend

### 2.6 Immunofluorescence staining

At the endpoint of the study, mice were sacrificed, and tumors were harvested and prepared for immunohistology staining. After deparaffinizing, antigen retrieval, and permeabilization, tissue sections were blocked in 1% bovine serum albumin (BSA) at room temperature for 1 h. Primary antibodies conjugated with fluorophores (Table 1) were incubated overnight at 4°C, and nuclei were counterstained with DAPI‐containing mounting medium (Vector Laboratories Inc., Burlingame, CA). All antibodies were diluted according to the manufacturer's manual. Fluorescence images were acquired using Zeiss 880 confocal laser scanning microscope.

### 2.7 Luminex assay

A multiplex analysis kit (Cytokine & Chemokine Convenience 26‐Plex Mouse ProcartaPlex™ Panel 1) for 26 chemokines and cytokines was obtained from Invitrogen (Waltham, MA). Tumor samples were homogenized by hard tissue homogenizing mix with bead homogenizer, then the assay performed according to the manufacturer's protocol. Data was collected using the Luminex‐MAGPIX® system (Luminex, Austin, TX). Data analysis was performed using the xPONENT software package (Luminex, Austin, TX). Five‐parameter regression was used to calculate the sample concentrations from the standard curves.

### 2.8 H&E staining

The tumors were fixed in 4% formalin, paraffin‐embedded, and sectioned for hematoxylin and eosin (H&E) staining. Metastasis was determined by H&E staining and photographed by optical microscopy.

### 2.9 Statistical analysis

All data are presented as mean ± SD. GraphPad Prism V.8 software was used to generate graphs and perform statistical analyses. Either Student's *t*‐test or two‐way analysis of variance (ANOVA) was used to determine statistically significant differences between treatment groups. Survival analysis was performed using the Kaplan–Meier method with a log‐rank test to determine statistical significance. All *p* values less than 0.05 were considered statistically significant.

## 3 RESULTS

### 3.1 S15 and TGF‐β are both upregulated in human breast cancer patients

**FIGURE 1:**
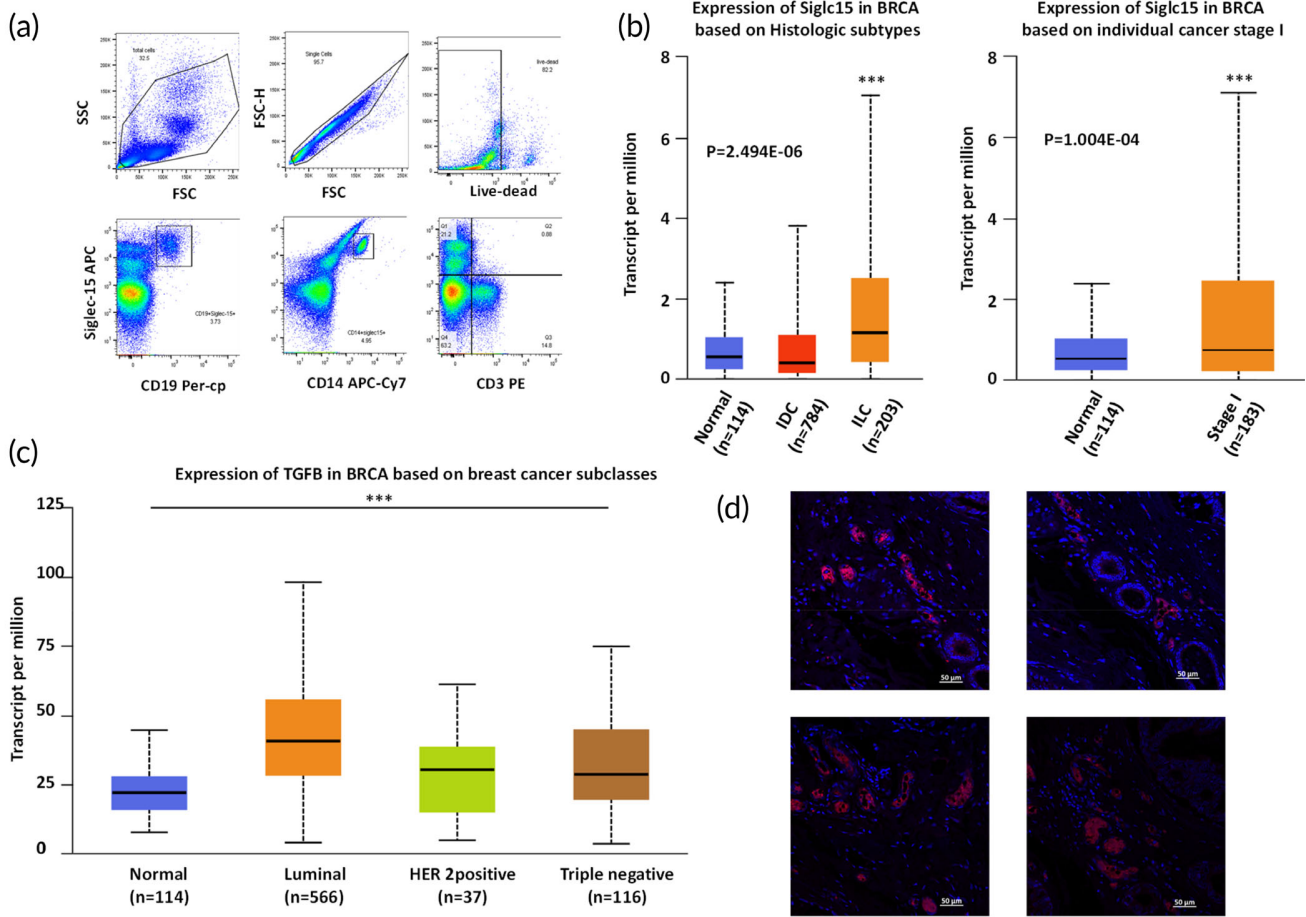
Expression and upregulation of S15 and TGF‐β in human PBMCs and breast cancer patient. (a) PBMCs were harvested from different donors and analyzed by flow cytometry. Single cell staining and FMO were performed (*n* = 3). (b) S15 are upregulated in breast cancer tumor tissue compared to normal tissue, based on TCGA database. (c) TGF‐β are upregulated in different classes of breast cancer tumor tissue compared to normal tissue. (TCGA data analysis, *n* = 1100). (d) Siglec15 expression was detected in triple negative breast cancer patient samples (*n* = 3).

We first investigated the expression of S15 on immune cells from human PBMCs. We detected S15 expression only on a subset of macrophages and B cells, with minimal expression on T‐cells (Figure 1a), in good agreement with previous findings.^9^ Next, utilizing the TCGA data bank, we confirmed that S15 (Figure 1b) and TGF‐β (Figure 1c) were upregulated in breast cancer tissue compared to normal tissue, the latter in both HER2‐positive and TNBC tissues. We then performed histology on tumor specimens from TNBC patients, and consistently detected S15 expression (Figure 1d). These results confirmed S15 is indeed present and likely upregulated in breast cancer tissues.

### 3.2 Construction and characterization of S15/TGF‐β bsAb

**FIGURE 2:**
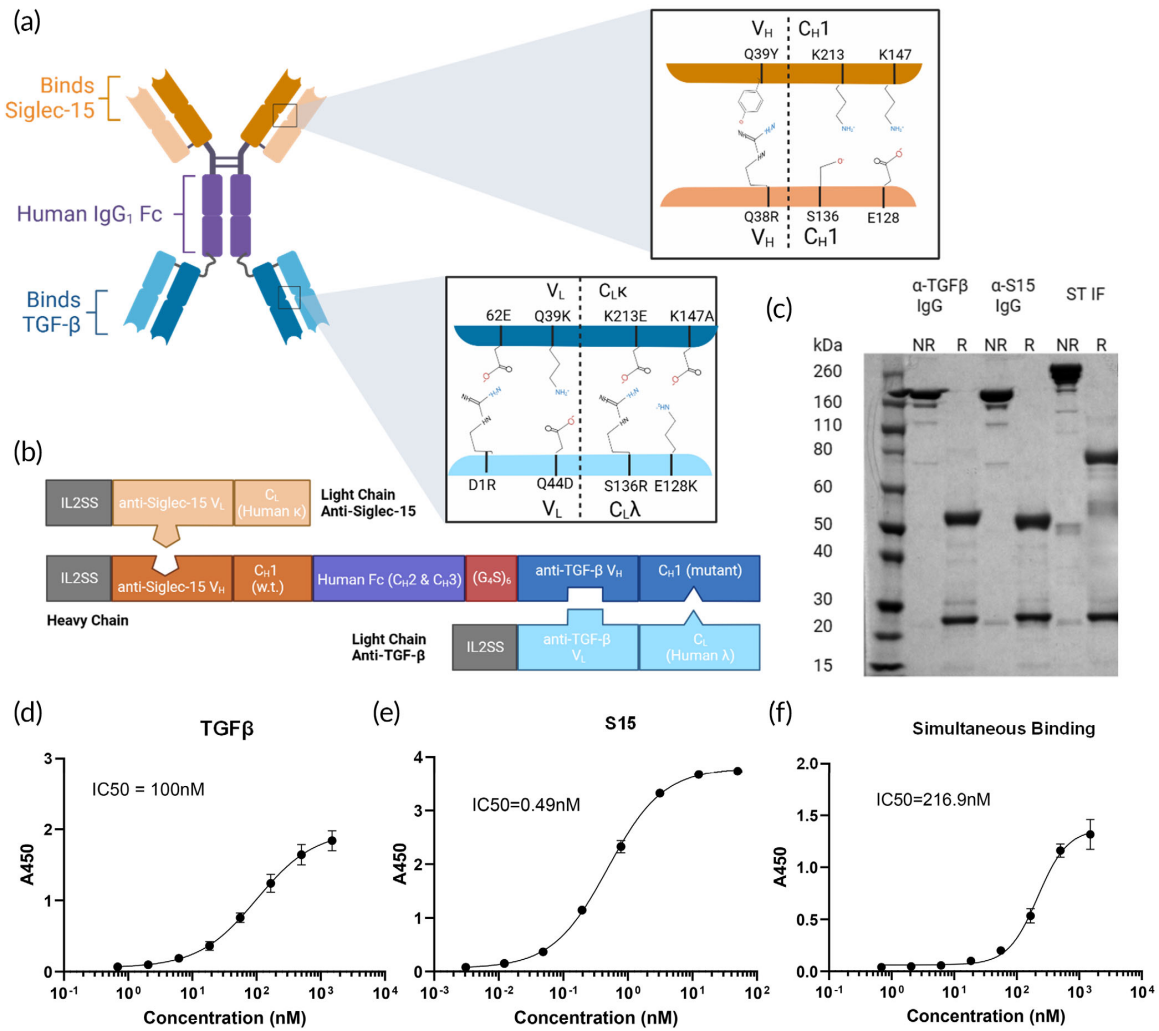
Characterization of a S15/TGF‐β bsAb. (a) Schematic of the structure of a IgG‐Fab that binds S15 and TGFβ. Orthogonal amino acid mutations, denoted using the Kabat numbering scheme, were introduced to ensure proper heavy‐ and light‐chain pairings. (b) Corresponding sequence map of the heavy chain and two light chains used to produce the Fab‐IgG through simple co‐expression. Created with Biorender.com (c) SDS–PAGE of parent IgGs and ST IF, either not reduced (NR) or reduced with TCEP (R). (d) ELISA binding curve of ST IF against TGFβ. (e) ELISA binding curve of ST IF against S15. (f) Simultaneous binding curve of ST IF in sandwich ELISA, using TGFβ coated plate and S15‐biotin and streptavidin‐HRP for detection.

We synthesized the S15/TGF‐β (ST) bsAb with a IgG‐Fab design, utilizing the OrthoMab platform that ensures proper pairing of distinct heavy and light chains by introducing a small number of site‐specific amino acid mutations in both the heavy chain and light chain at the CH1 interface (Figure 2a,b). ST bsAb displayed the expected size (Figure 2c), and bound both S15 (Figure 2d) and TGF‐β with nanomolar affinity (Figure 2e). To confirm that ST simultaneously binds both S15 and TGF‐β, we performed a sandwich ELISA in which ST was captured by a TGFβ coated plate, incubated with biotinylated S15, then detected with streptavidin HRP. The simultaneous binding affinity was ~217 nM (Figure 2f).

**FIGURE 3:**
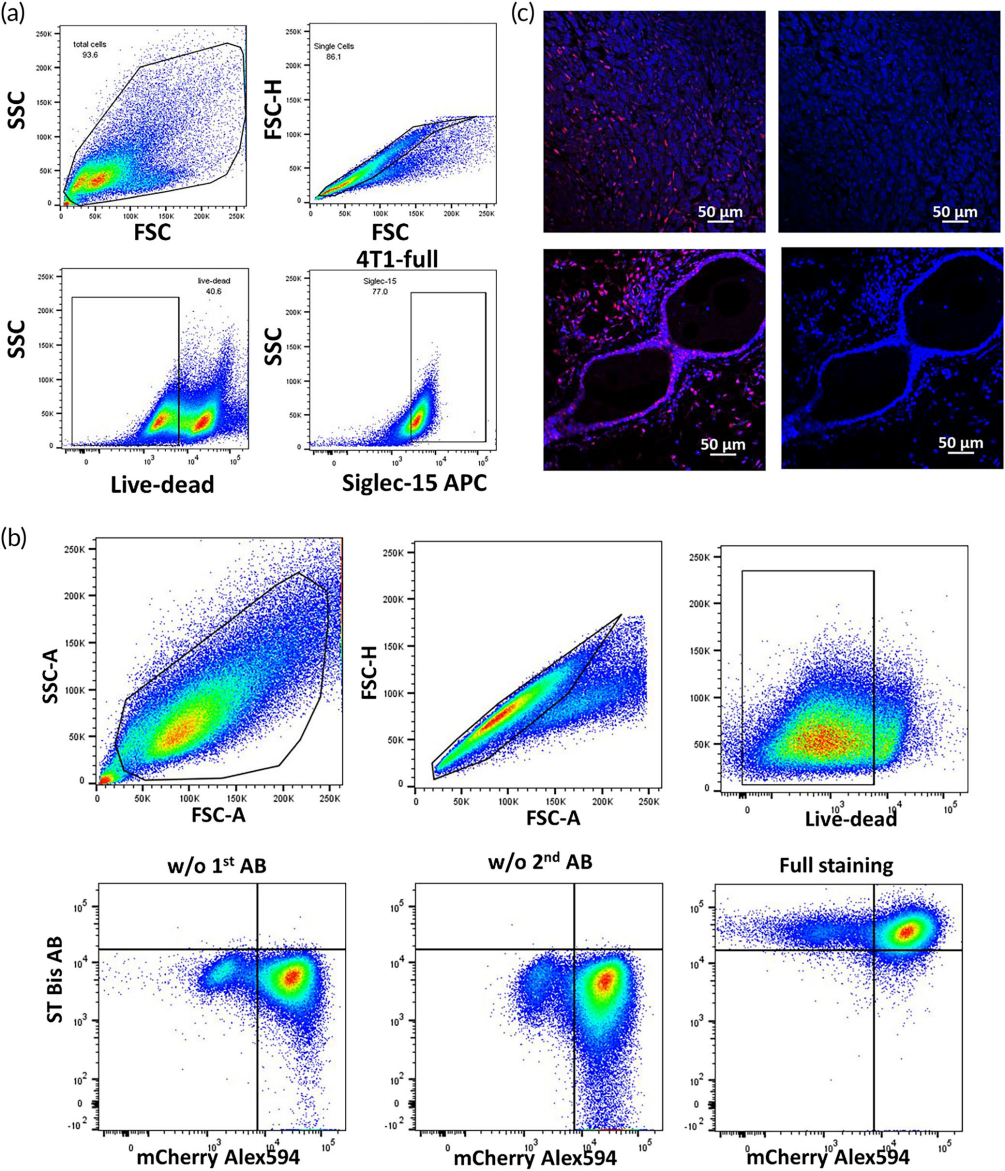
Detection of S15 expression in vitro on 4T1 tumor cells and binding of siglec15‐TGF‐β bsAb in mouse 4T1 triple negative breast tumor cells. (a) 4T1 cells from cell culture were analyzed with anti‐siglec 15 mAb by flow cytometry. (b) Tumors from mice inoculated with 4T1 cells orthotopically for 14 days were harvested, and single cell suspension were stained with ST bsAb. FMO were performed (*n* = 5). (c) Tumor tissues were harvested from mice with orthotopic 4T1 tumors, and stained for Siglec15 in Alex488 (red). Negative staining and control staining were performed.

To ascertain that ST bsAb can bind murine 4T1 TNBC, as well as the suitability of the model to test for effect of S15 targeting, we first performed flow cytometry of 4T1 with labeled anti‐S15 mAb, and detected high levels of S15 expression (Figure 3a). We saw similar specific binding of ST against 4T1 (Figure S1), modest binding to dendritic cells and macrophages that were previously found to possess low levels of surface S15 expression, and minimal binding to cells that are negative for S15 such as B‐ and T‐cells (Figure S2). We next isolated 4T1 cells from mice bearing orthotopic 4T1 tumors, and found that the ST bsAb effectively bound the isolated 4T1 cells (Figure 3b). We also performed histology on tumor tissues collected from the 4T1 tumor bearing mice, and similarly saw extensive binding of engineered ST bsAb bound to tumor tissues (Figure 3c).

### 3.3 ST bsAb inhibited the growth of established 4T1 TNBCs in mice

**FIGURE 4:**
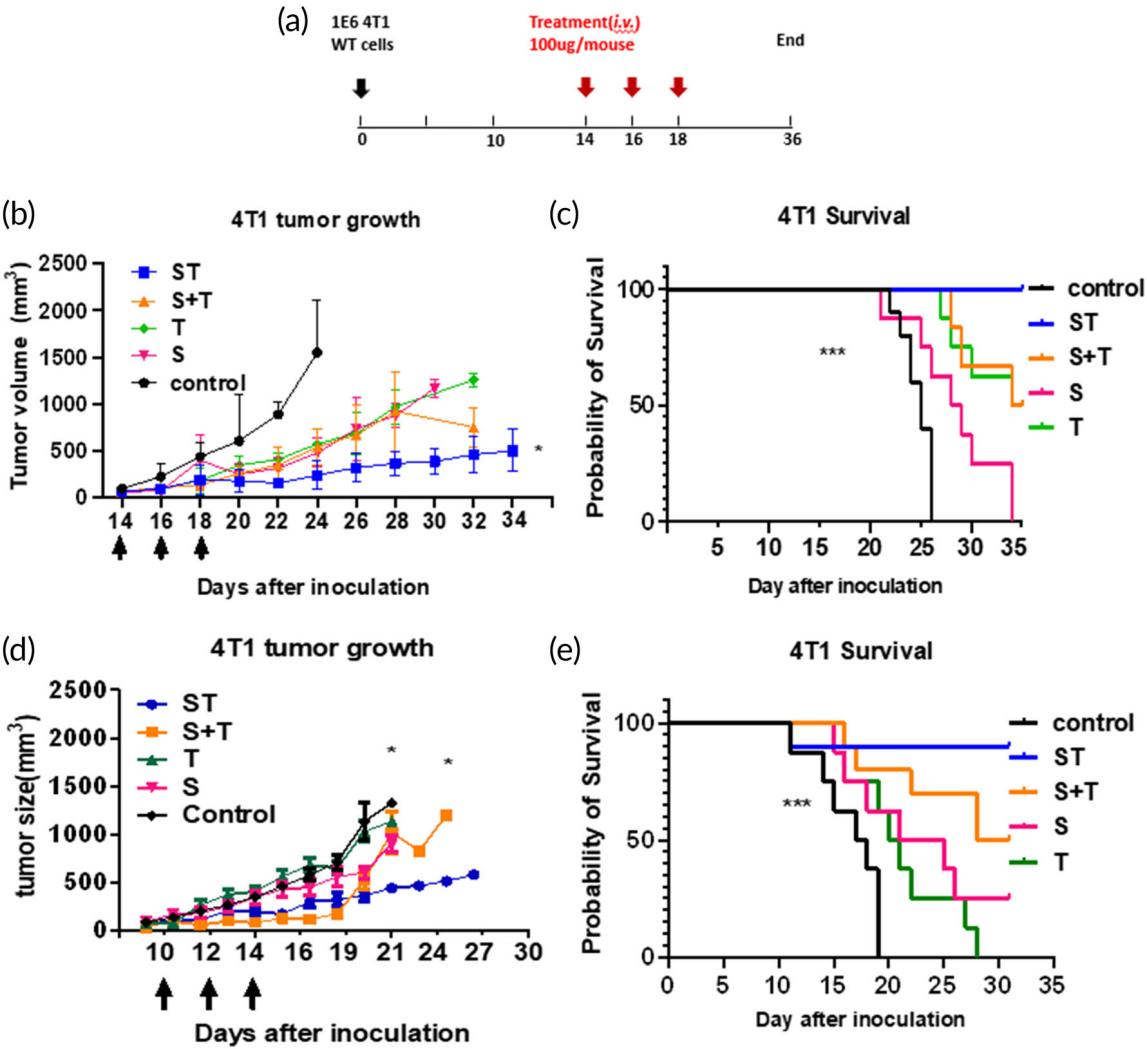
Siglec15‐TGF‐β (ST) bsAb effectively inhibited 4T1 tumor growth and markedly prolonged overall survival compared to mAb against S15 (S), TGF‐β (T), and cocktail of both S15 and TGF‐β (S + T) mAbs. Tumor inhibition and host survival after ST bsAb in 4T1 triple negative breast cancer tumor models. The tumor‐bearing mice were divided into five groups and treated with either control Ab, ST, S, T, and S + T. (a) Study timeline. (b and d) Tumor growth inhibition in mice with orthotopic 4T1 tumors. Arrows indicate the time of treatment injection. (c and e) The Kaplan–Meier survival curves of the variants treated groups. Each group comprised of 5–8 mice (in figure (b) control: *n* = 10, ST: *n* = 8, S + T: *n* = 6, S: *n* = 8, T: *n* = 8; in figure (e) control: *n* = 8, ST: *n* = 10, S + T: *n* = 10, S: *n* = 8, T: *n* = 8). Arrows indicate the days mice received IV injection of different mAbs. *p < 0.05, **p < 0.01, and ***p < 0.001. The statistical analyses were calculated through comparison with the control group.

We proceeded to assess the efficacy of ST bsAb therapy in the 4T1 TNBC murine model. On Day 14 following inoculation of the 4T1 tumors, mice were given various treatments, administered a total of three times given every 2 days (Figure 4a). The treatments included the bsAb ST, a control mAb, a mAb against S15 only (S), a mAb against TGF‐β only (T), and a mAb cocktail of both S and T (S + T). We were able to observe clear evidence of tumor suppression with all of the non‐control mAbs, with apparent anti‐tumor activity following the very first of the three doses (Figure 4b). The overall anti‐tumor activity of S, T, and S + T were similar across the entire duration of the study, with slowed but sustained growth of the tumor (Figure 4b). All three mAb treatment resulted in superior survival compared to control mAb, and had similar overall survival between the different mAbs, with all mice passing the point necessitating humane sacrifice by Day 34 (Figure 4c).

In contrast to these interventions, the ST bsAb treatment led to more effective inhibition of tumor growth (Figure 4b, Figure S3), with statistically significant suppression of the tumor compared to all other mAb treatments (*p* < 0.05), and no statistically significant difference in the tumor size within the ST bsAb treatment group between the last day of the treatment (Day 18) through the end of the study (Day 36). All mice receiving the ST‐treatment survived past Day 35 (Figure 4c). To ensure the reproducibility of our findings, we executed an independent but otherwise identical study, and observed virtually the same enhanced tumor suppression and survival benefit following ST‐treatment versus the other interventions (Figure 4d,e).

**FIGURE 5:**
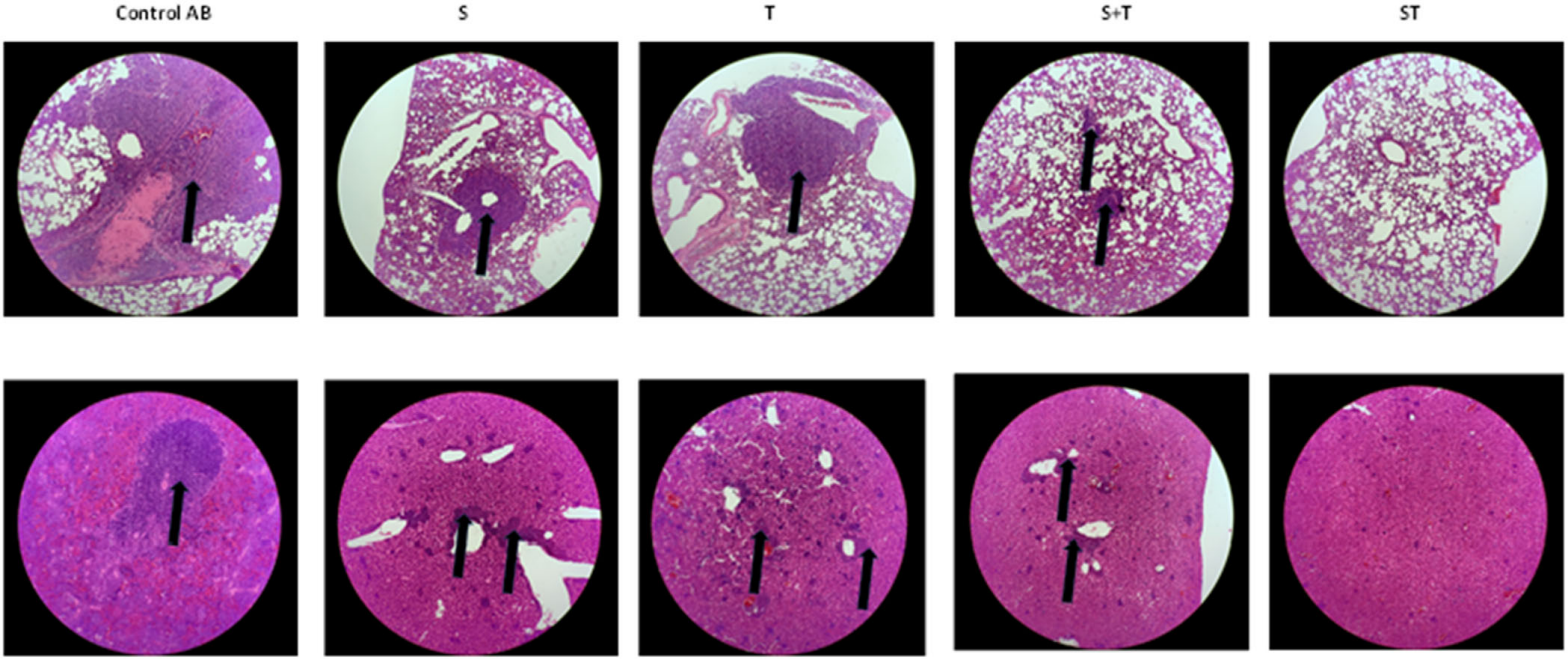
H&E staining of major organs in each group. Upper channel were lung and lower channel are the liver from tumor bearing mice from each group. The black arrows showed tumor metastasis.

We wished to further evaluate whether various treatments could induce sufficient anti‐tumor immunity that impact TNBC metastasis. Thus, at the end of the study, we also collected lung and liver tissues for histology; lung and liver are two of the most common organs of metastasis found in TNBC. In animals treated with control mAb, we were able to readily detect large metastasis in both the lung and liver (Figure 5), in good agreement with prior studies. In animals receiving monospecific mAbs (S, T, or S + T), we were also able to consistently detect lung and liver metastasis in all the animals, although the size of the metastasis appeared to be much smaller than those in animals treated with control mAb. Interestingly, no metastasis was detected in either the lung nor the liver in all the mice treated with the ST bsAb (Figure 5). ImageJ‐based quantitation of various histology images is provided in Figure S4. These results indicate that, in addition to effective suppression of the growth of the primary tumor, the ST bsAb may also effectively reduce 4T1 metastasis, either by reducing the invasiveness and metastatic potential of the primary tumor, or by facilitating effective killing of the metastasis.

### 3.4 ST bsAb modulated the immune‐suppressive TME

**FIGURE 6:**
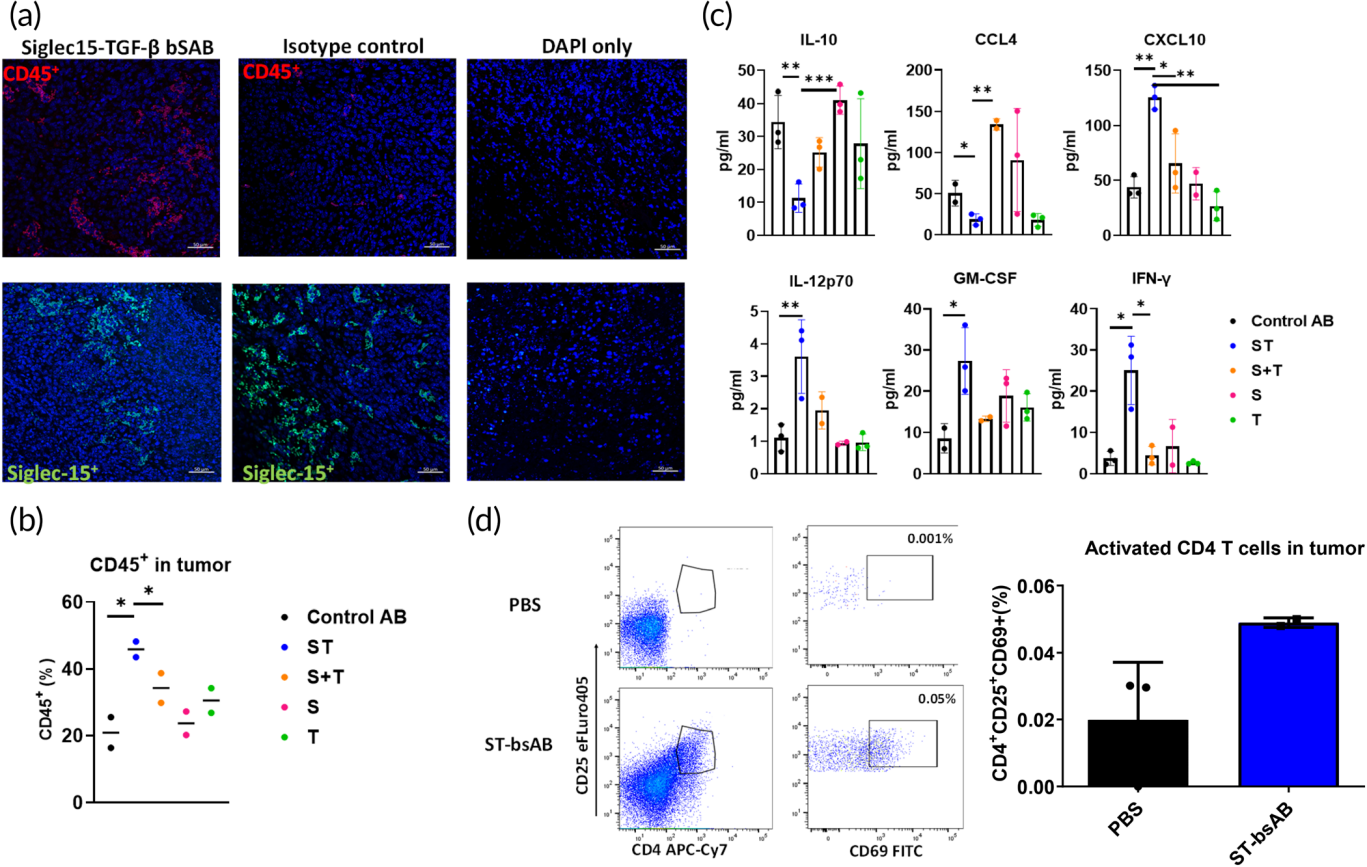
ST bsAb reduced immunosuppression within the TME. (a) ST treatment enhanced the presence of CD45+ cells while reducing the expression of S15 in TME. Tumor tissues were harvested at the end of the experiment. The sections were stained by CD45+ Alex594 (red) and Siglec15 Alex488 (green). Negative staining and control staining were performed. (b) ST treatment increased CD45+ cells in tumor, as determined by flow cytometry of tumor‐derived single cell suspension. Single staining and FMO were performed (*n* = 5). (c) Expression of various cytokines and chemokines confirmed more effective inhibition of immune suppression by ST versus S, T, and S + T treatment. Tumor tissue was harvested and homogenized with RIPI buffer. Tumor lysates were collected, and various cytokines/chemokines were quantified using Luminex. (d) ST treatment increased activated CD4+ T‐cells in the TME, assessed by flow cytometry of tumor‐derived single cell suspension. Single staining and FMO were performed (*n* = 5).

Tumor growth is determined not only by intrinsic tumor biology, but also by the immune microenvironment within the tumor. To determine what factors may substantiate the more effective tumor growth suppression observed with the bsAb ST, we next assessed whether the treatment altered specific immunological aspects of the TME. We first focused on characterizing the immune cell populations. CD45 is an important regulator of T‐ and B‐cell antigen receptor signaling; activated, memory and cytotoxic tumor‐infiltrating T‐lymphocytes all express CD45 on their cell surface.^24^ We were able to consistently detect a marked increase in the CD45+ cells in tumors of mice treated with ST, as revealed by both staining of tumor sections (Figure 6a) as well as flow cytometry analysis of tumor‐derived single cell suspensions (Figure 6b) compared to the different treatment groups. Beyond an increase in CD45+ cells overall and activated CD4+ T cells, we also found an increase in CD4+ T cells, a marked reduction in Bregs, an increase in CD11c+ dendritic cells, and a decrease in the ‘tolerant’ M2 macrophages in mice receiving ST bsAb (Figure S5). These data suggest that ST bsAb may potentially induce a holistic, multifaceted immune response against the cancer cells within the TME.

Beyond cells, the TME is also characterized by the secretion of diverse cytokines in different types and stages of cancers, capable of either inhibiting tumor growth or contributing to chronic inflammation that supports tumor growth in both autocrine and paracrine manners leading to poor cancer outcomes. We thus next assessed the change in cytokines in the TME following treatment with different mAbs. Consistent with the enhanced tumor suppression following ST bsAb treatment, the level of proinflammatory cytokines such as IL‐10 and CCL4 that inhibit anti‐tumor immunity was significantly decreased (Figure 6c). The chemokine CXCL10 is a seven trans‐membrane‐spanning G protein‐coupled receptor^25^ predominantly expressed on activated T lymphocytes (Th1), natural killer (NK) cells, inflammatory dendritic cells, macrophages, and B cells,^25^, ^26^ and serves as a chemoattractant for monocytes, T cells, and NK cells. We found markedly increased CXCL10 in mice treated with ST but not in mice treated with other mAbs (Figure 6c). GM‐CSF is essential for the differentiation of dendritic cells responsible for processing and presenting tumor antigens for the priming of antitumor cytotoxic T lymphocytes.^27^ IL‐12 regulates inflammation by linking innate and adaptive immune responses,^28^ generally through increased secretion of interferon‐γ (IFN‐γ) that promote Th1 cell immunity and stimulate M1 macrophage as part of an overall activation of anti‐tumor immune response.^29^ Consistent with reduced immune suppression, treatment with ST bsAb significantly enhanced GM‐CSF secretion, increased IL‐12p70, and increased IFN‐γ levels in the TME compared to all other mAb treatments (Figure 6c). Finally, we saw increased activated CD4 T cells in mice treated with ST (Figure 6d). Together, these results underscored the effectiveness of the ST bsAb in mediating a robust, multifaceted reduction in immunosuppression that culminated in effective tumor suppression.

### 3.5 ST bsAb enhanced systemic T cell activation

**FIGURE 7:**
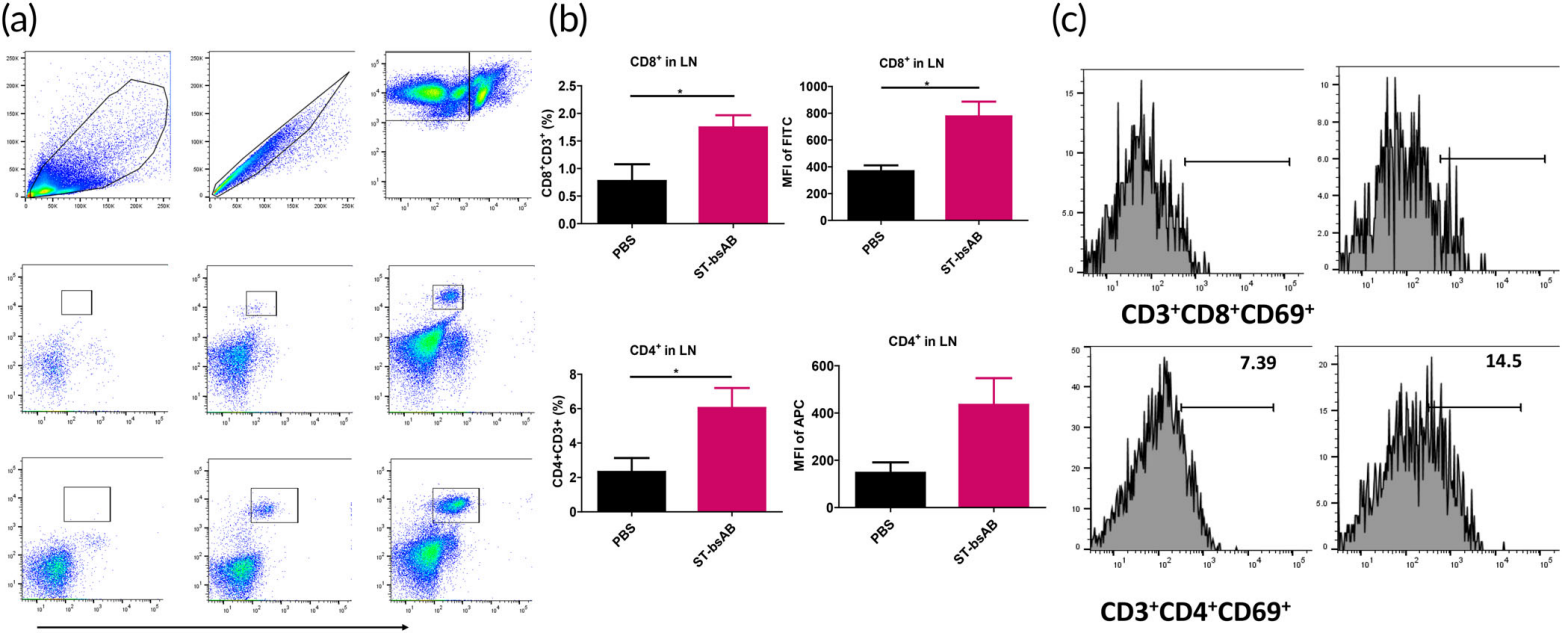
ST bsAb treatment enhanced systemic anti‐tumor T cell response. (a and b) Increased T‐cells in LN from ST‐treated mice over vehicle control. (c) Following bsAb treatment, we detect increased activated CD4^+^ and CD8^+^ T‐cells in LN. LN were harvested, and single cell suspension were isolated for flow cytometry. Single staining and FMO were performed (*n* = 5).

Recent studies have shown that systemic activation of T cells, reflected by patients with higher numbers of circulating CD4+ and CD8+ T‐cells, was associated with an overall survival benefit compared to patients with a low number of circulating CD4+ T‐cells. We thus analyzed the number of CD4+ and CD8+ T‐cells in lymph nodes from tumor bearing mice following different treatments. Following ST treatment, we were able to detect markedly more CD4^+^ and CD8^+^ T‐cells (Figure 7a,b), including activated CD4^+^ and CD8^+^ T‐cells (Figure 7c). We also found a statistically significant increase in CD4+ and CD8 T cell populations in the lymph nodes of animals treated with ST bsAb, including both activated CD4 and CD8 T cells (Figure S6). These results are consistent with ST bsAb mediating an active, ongoing immune response in 4T1‐TNBC bearing mice, and may be responsible for the impressive reduction in liver and lung metastasis observed with the ST bsAb treatment.

## 4 DISCUSSION

Over the past decade, cancer immunotherapy has undergone a marked paradigm shift, from systemic activation of immune responses via immune enhancement^4^, ^30^ to selective correction of defective immunity in the TME via immune normalization.^31^ Unfortunately, tumors can evade host immune surveillance through multiple distinct mechanisms,^32^ and it is also difficult to sustain effective concentrations of immunomodulators in the TME. Not surprisingly, the vast majority of TNBC patients failed to respond to monospecific immunomodulatory therapies in clinical trials, including those targeting PD‐1/PD‐L1 or CTLA4. The objective response rate, which measures the percentage of patients whose disease decreased (i.e., partial response) and/or disappeared (complete response) after treatment, is typically only ~20%.^33^, ^34^ Furthermore, although combined immunotherapy and chemotherapy appeared promising in early trials such as Impassion130^35^, ^36^ and KEYNOTE‐355^37^, ^38^ culminating in the FDA approval of Tecentriq in 2019 and Keytruda in 2020 for TNBC, some of the observed efficacy was not reproduced in later clinical trials. For instance, Tencentriq failed to improve progression free survival and overall survival compared to paclitaxel alone,^39^ leading to its voluntary withdrawal in 2021. These developments strongly underscore the need of additional methods that could effectively normalize the immune TME in TNBC.

As discussed in Section 1, we believe the ideal TME immunomodulatory intervention should: (1) target an antigen that is highly expressed on tumor, with minimal expression on healthy tissue, thereby enabling highly selective accumulation in the target tumor; (2) inhibit tumor induced immunosuppression of antigen presenting cells and T cell function within TME. Here, taking advantage of S15 both as a cellular target of TNBC cells as well as an immunomodulatory target, we engineered a bsAb that targets both S15 and TGF‐β. To our surprise, this ST bsAb was highly effective at suppressing tumor growth leading to a clear survival benefit, but also appeared to reduce immunosuppression within the tumor and normalize the dysregulated anti‐tumor response. Importantly, the effectiveness of suppressing tumor growth and reducing immunosuppression with the ST bsAb was far greater than targeting S15 alone, targeting TGF‐β alone, and even a cocktail of both S and T mAbs, underscoring the importance of covalently linking the S15 and TGF‐β binding moieties, presumably due to greater accumulation of TGF‐β binding moieties within the tumor. These results both support our framework for designing therapeutic molecules that can effectively modulate the immunity in the TME, and S15 as a potential target for developing candidate molecules that could modulate the tumor immune microenvironment and enable more effective cancer immunotherapy.

S15 was first discovered by Lieping Chen's group as a critical immune suppressor of T‐cells via genome‐scale T‐cell activity array.^40^ In their landmark study, S15 expression was reported for lung, kidney, bladder, and liver cancers, among various cancer tissues tested; however, TNBC cells were not evaluated. Here, we not only confirmed that S15 is highly expressed in TNBCs, but that targeting S15 is essential for the observed tumor suppression by the ST bsAb, as well as effective reduction of a variety of immunosuppressive responses. Given that S15 has limited expression in normal tissues and immune cells, and is not an essential molecule for the normal survival and organ development, we believe a S15 blockade strategy is likely safe.

TGF‐β is a multifunctional cytokine involved in both induction of Tregs and inhibition of cytotoxic T cells in the TME, inhibition of the activity of TAMs, promotion of M1‐to‐M2 phenotype polarization, and induction of PD‐L1 up‐regulation.^41^, ^42^, ^43^ Given its multifaceted function, and ability to augment ROR1‐specific CAR‐T for TNBC,^44^ it served as an obvious first target for our bsAb engineering, as had been exploited by others. Nevertheless, mAbs against TGF‐β have not been effective clinically, presumably because TGF‐β binding alone would not confer sustained high levels in the TME. Indeed, since TGF‐β is a soluble growth hormone that is ubiquitously present throughout the body, an anti‐TGF‐β mAb is unlikely to be preferentially retained within the tumor milieu, even if the mAb extravasates from the tumor vasculature. Instead, the concentration of an anti‐TGF‐β mAb is unlikely to be elevated within the tumor compared to elsewhere in the body. In turn, any anti‐TGF‐β mAb treatment is unlikely to sufficiently bind and saturate free TGF‐β within the tumor to the extent necessary to reduce tumor growth and invasiveness, consistent with our findings here in animals treated with anti‐TGF‐β only mAb. By binding to S15 on the TNBC cells surface, it is likely our ST bsAb are able to accumulate within the TNBC tumor to a greater extent than anti‐TGF‐β mAb can, thus resulting in greater TGF‐β binding.

Finally, bsAb continues to be gaining increasing attention for the treatment of TNBC^45^ and various other cancers,^46^, ^47^ with a number of bsAb targeting PD‐L1 and TGF‐β currently in mid‐to‐late clinical trials for multiple cancers.^48^, ^49^, ^50^ Unfortunately, their results have been disappointing to date. While in principle very similar to our ST bsAb format, we believe the limited efficacy is rooted in the fact that TNBC appears to readily evade PD‐L1 blockage, given the modest efficacy with PD‐L1 targeting to date.^33^, ^34^ When that happens, a PD‐L1/ TGF‐β bsAb would also fail to achieve preferential accumulation or prolonged retention in the tumor, and thus could only afford transient alleviation of the overall strong immunosuppression within the TNBC‐TME. It remains to be seen whether TNBC can readily evade S15‐binding in clinical studies.

## AUTHOR CONTRIBUTIONS

**Limei Shen:** Conceptualization (lead); formal analysis (lead); investigation (lead); methodology (lead); project administration (equal); supervision (equal); visualization (equal); writing – original draft (lead); writing – review and editing (equal). **Alison M. Schaefer:** Formal analysis (supporting); investigation (supporting); methodology (supporting); visualization (equal); writing – review and editing (supporting). **Karthik Tiruthani:** Conceptualization (supporting); investigation (supporting); methodology (equal). **Whitney Wolf:** Investigation (supporting). **Samuel K. Lai:** Conceptualization (equal); formal analysis (supporting); funding acquisition (lead); project administration (equal); supervision (lead); writing – original draft (supporting); writing – review and editing (lead).

## FUNDING INFORMATION

This work was financially by National Institutes of Health, through Grant Award Number R21CA273983 (Samuel K. Lai); Lung Cancer Initiative of North Carolina (Limei Shen); and startup funds from UNC School of Pharmacy (Samuel K. Lai). The content is solely the responsibility of the authors and does not necessarily represent the official views of the NIH and other funders.

## CONFLICT OF INTEREST STATEMENT

Samuel K. Lai, Limei Shen, Alison M. Schaefer, and Karthik Tiruthani are inventors of submitted patent application focused on S15‐targeted bispecific antibodies for cancer applications. The terms of these arrangements are managed by UNC‐CH in accordance with its conflict‐of‐interest policies.

### PEER REVIEW

The peer review history for this article is available at https://www.webofscience.com/api/gateway/wos/peer-review/10.1002/btm2.10651.

## Supporting information


**FIGURE S1.** Detection of ST binding against S15^+^ 4T1mCherry cancer cells.
**FIGURE S2.** Detection of ST binding with different splenocytes from mouse. Splenocytes were isolated from C57BL/6 mice, and single cell suspension was prepared. Flow cytometry was performed following ST incubation and after staining. As expected, ST exhibited modest binding to dendritic cells (CD11c+) and macrophages(F4/80+), both of which were previously found to express modest levels of S15, but otherwise showed no detectable binding to T cells and B cells that are S15‐negative.
**FIGURE S3.** Tumor inhibition after ST bsAb treatment in 4T1 triple negative breast cancer tumor models. The tumor‐bearing mice were divided into 5 groups and treated with either control Ab, ST, S, T, and S + T.
**FIGURE S4.** Quantitative analysis of HE histological staining for metastasis in lung and liver. Analysis was performed by ImageJ.
**FIGURE S5.** (a–d) Immune cells in the tumor at the time of sacrifice in mice treated with ST versus saline: (a) activated CD4+ T cells, (b) activated CD8+ T cells, (c) regulatory B cells, and (d) monocytes and macrophages. ST induces (e) a marked increase in the MHC (major histocompatibility complex) II+ monocyte‐like cells, (f) a marked decrease in M2 macrophages, and (g) a marked decrease in Bregs.
**FIGURE S6.** Detection of activated T cells in LN in tumor bearing mice.

## Data Availability

The data that support the findings of this study are available from the corresponding author upon reasonable request.
