# Supplementary material for: Siglec15/TGF‐β bispecific antibody mediates synergistic anti‐tumor response against 4T1 triple negative breast cancer in mice
Source: Bioeng Transl Med. 2024 Mar 11;9(5):e10651. doi: 10.1002/btm2.10651 (PMC11561775; doi:10.1002/btm2.10651)
Supplement: Supplementary file 1 — FIGURE S1. Detection of ST binding against S15+ 4T1mCherry cancer cells. FIGURE S2. Detection of ST binding with different splenocytes from mouse. Splenocytes were isolated from C57BL/6 mice, and single cell suspension was prepared. Flow cytometry was performed following ST incubation and after staining. As expected, ST exhibited modest binding to dendritic cells (CD11c+) and macrophages(F4/80+), both of which were previously found to express modest levels of S15, but otherwise showed no detectable binding to T cells and B cells that are S15‐negative. FIGURE S3. Tumor inhibition after ST bsAb treatment in 4T1 triple negative breast cancer tumor models. The tumor‐bearing mice were divided into 5 groups and treated with either control Ab, ST, S, T, and S + T. FIGURE S4. Quantitative analysis of HE histological staining for metastasis in lung and liver. Analysis was performed by ImageJ. FIGURE S5. (a–d) Immune cells in the tumor at the time of sacrifice in mice treated with ST versus saline: (a) activated CD4+ T cells, (b) activated CD8+ T cells, (c) regulatory B cells, and (d) monocytes and macrophages. ST induces (e) a marked increase in the MHC (major histocompatibility complex) II+ monocyte‐like cells, (f) a marked decrease in M2 macrophages, and (g) a marked decrease in Bregs. FIGURE S6. Detection of activated T cells in LN in tumor bearing mice. [file BTM2-9-e10651-s001.docx]

**Supplementary data**


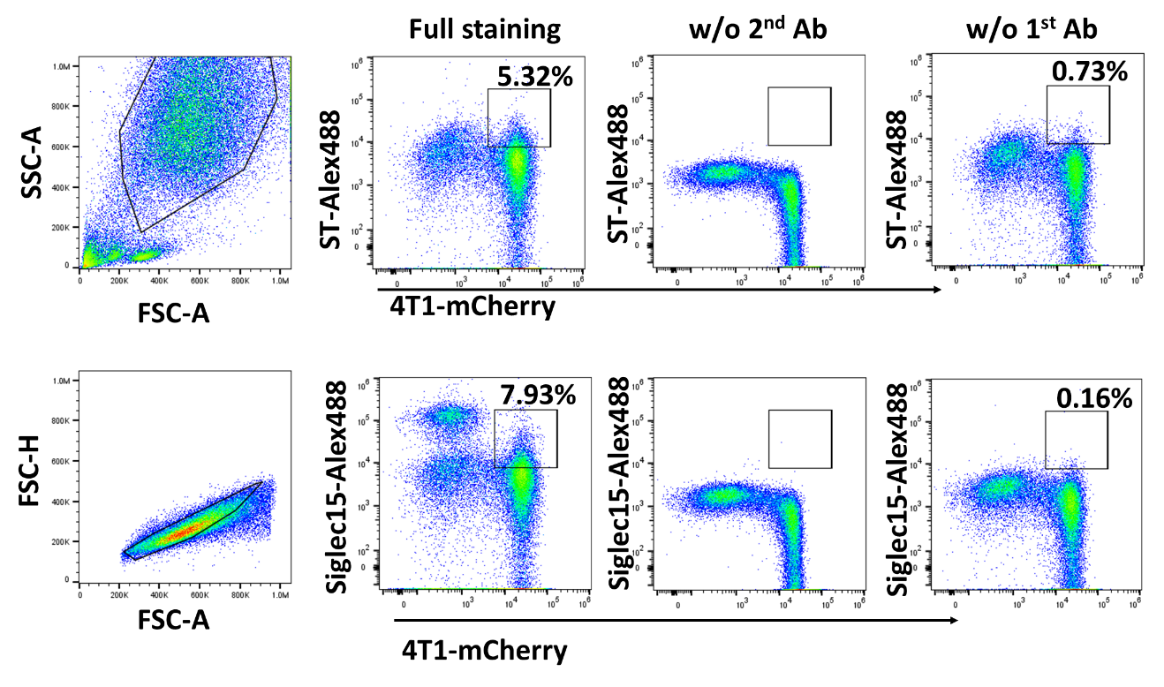


**Supplementary Figure S1.** Detection of ST binding against S15^+^ 4T1mCherry cancer cells.

**Supplementary Figure S2.** Detection of ST binding with different splenocytes from mouse. Splenocytes were isolated from C57BL/6 mice, and single cell suspension was prepared. Flow cytometry was performed following ST incubation and after staining. As expected, ST exhibited modest binding to dendritic cells (CD11c+) and macrophages(F4/80+), both of which were previously found to express modest levels of S15, but otherwise showed no detectable binding to T cells and B cells that are S15-negative.

**Supplementary Figure S3.** Tumor inhibition after ST bsAb treatment in 4T1 triple negative breast cancer tumor models. The tumor-bearing mice were divided into 5 groups and treated with either control Ab, ST, S, T, S+T.


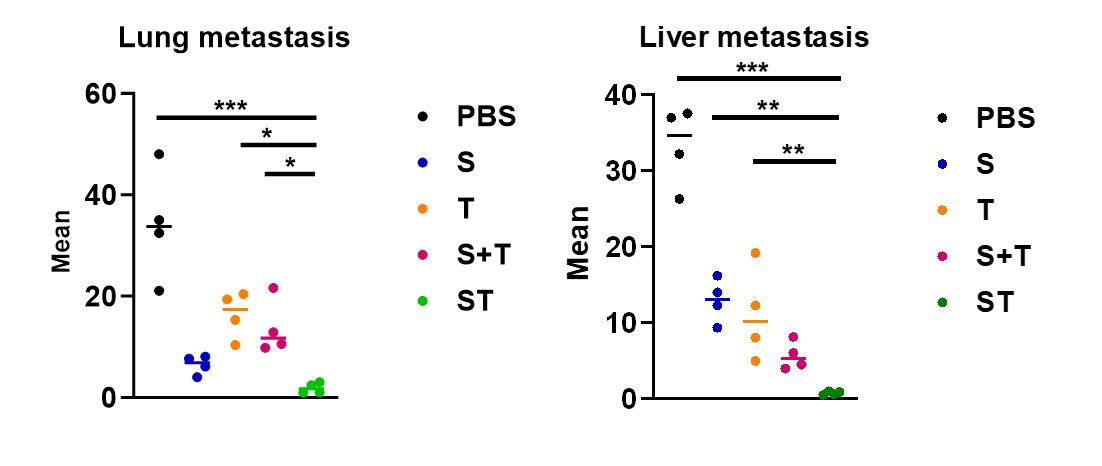


**Supplementary Figure S4.** Quantitative analysis of HE histological staining for metastasis in lung and liver. Analysis was performed by ImageJ.


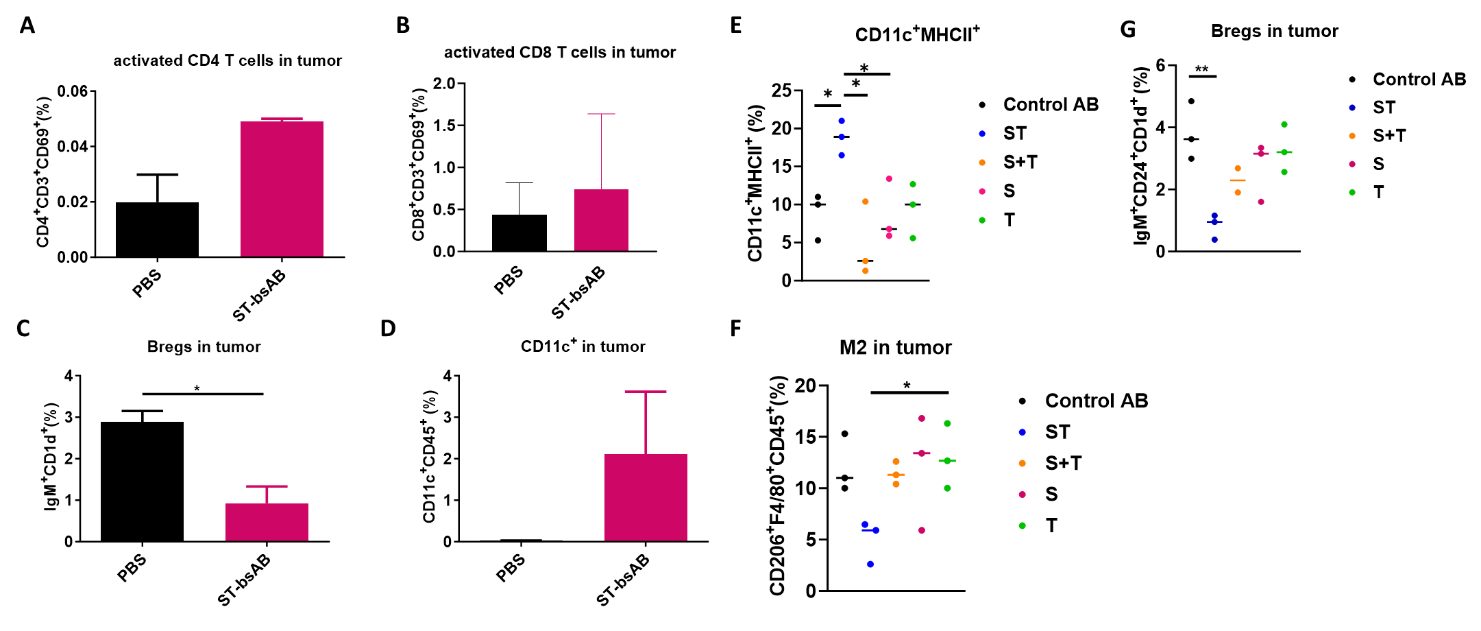


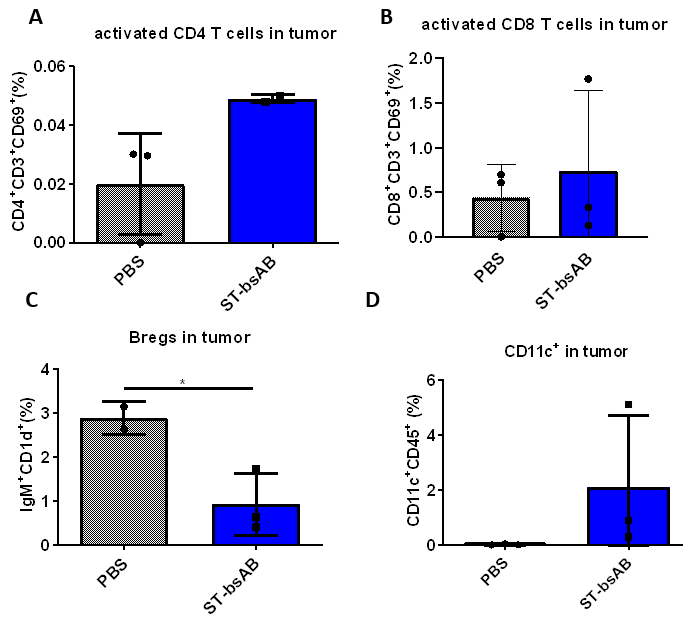


**Supplementary Figure S5. (A-D)** Immune cells in the tumor at the time of sacrifice in mice treated with ST vs. saline: **(A)** activated CD4+ T cells, **(B)** activated CD8+ T cells, **(C)** regulatory B cells and **(D)** monocytes and macrophages. ST induces **(E)** a marked increase in the MHC (major histocompatibility complex) II+ monocyte-like cells, **(F)** a marked decrease in M2 macrophages, and **(G)** a marked decrease in Bregs.

**Supplementary Figure S6**. Detection of activated T cells in LN in tumor bearing mice.
